# Supplementary material for: Distinct Amphibian Elevational and Seasonal Phylogenetic Structures Are Determined by Microhabitat Variables in Temperate Montane Streams
Source: Animals (Basel). 2022 Jun 29;12(13):1673. doi: 10.3390/ani12131673 (PMC9264966; doi:10.3390/ani12131673)
Supplement: Supplementary file 1 [file animals-12-01673-s001.zip › animals-1746813-supplementary.pdf]

animals

MDPI

Supplementary Materials

Distinct Amphibian Elevational and Seasonal Phylogenetic Structures Are Determined by Microhabitat Variables in Temperate Montane Streams

Xi-Wen Peng [1,†], Jing Lan [1,2,†], Zi-Jian Sun [1,2], Wen-Bo Zhu [2,3] and Tian Zhao [1,2,*]

1 College of Fisheries, Southwest University, Chongqing 400715, China; pxw@swu.edu.cn (X.-W.P.); lan97hhh@outlook.com (J.L.); sunzj19@outlook.com (Z.-J.S.)
2 CAS Key Laboratory of Mountain Ecological Restoration and Bioresource Utilization & Ecological Restoration Biodiversity Conservation Key Laboratory of Sichuan Province, Chengdu Institute of Biology, Chinese Academy of Sciences, Chengdu 610041, China; wenbo_zhu2022@163.com
3 Central South Inventory and Planning Institute of National Forestry and Grassland Administration, Changsha 410014, China
* Correspondence: zhaotian@cib.ac.cn; Tel.: +86-(0)28-82-89-09-35
† These authors contributed equally to this work.

Table S1. Geographic information of the transects in the study area.

Transects	Elevation（m）	Longitude（E）	Latitude（N）
Banihu	390.5	110.0540	29.6825
Shuitianba	467	110.0433	29.6686
Dujiazui	475	110.0664	29.6891
Nanmuping	520	110.0478	29.7419
Chewan	978	110.0553	29.7594
Gongtongwan	1365	110.0916	29.7894
Sanbaice	1368	110.0564	29.7685
Miaowan	1388	110.0672	29.7731
Daping	1400	110.1111	29.7992
Dapingcunchitang	1401	110.1236	29.7925
Xiaozhuangping	1411.5	110.0977	29.7831
Yangjiangping	1429	110.0944	29.7860
Futianya	1492	110.1214	29.7878

Table S2. Amphibian species (and the abbreviations) that were detected in the study area.

Oder	Family	Species	Abbreviation
Anura	Ranidae	*Amolops chunganensis*	Amc
		Amolops sinensis	Ams
		Odorrana margaretae	Odm
		Odorrana schmackeri	Ods
		Odorrana yizhangensis	Ody
		Pelophylax nigromaculatus	Pen
		Rana jiemuxiensis	Raj
		Pseudorana sangzhiensis	Pss
	Rhacophoridae	*Polypedates egacephalus*	Pom
		Zhangixalus chenfui	Zhc
		Zhangixalus dennysi	Zhd
		Zhangixalus nigropunctatus	Zho
		Zhangixalus omeimontis	Zhs
	Dicroglossidae	*Quasipaa boulengeri*	Qub
		Fejervarya multistriata	Fem
		Feirana quadranus	Feq
	Bufonidae	*Bufo gargarizans*	Bug
	Hylidae	*Hyla gongshanensis wulingensis*	Hyg
	Megophryidae	*Leptobrachium boringii*	Leb
		Leptobrachella oshanensis	Leo
		Megophrys sangzhiensis	Mes
		Megophrys tuberogranulata	Met
	Microhylidae	*Microhyla fissipes*	Mif
		Microhyla heymonsi	Mih
Caudata	Hynobiidae	*Pseudohynobius flavomaculatus*	Psf

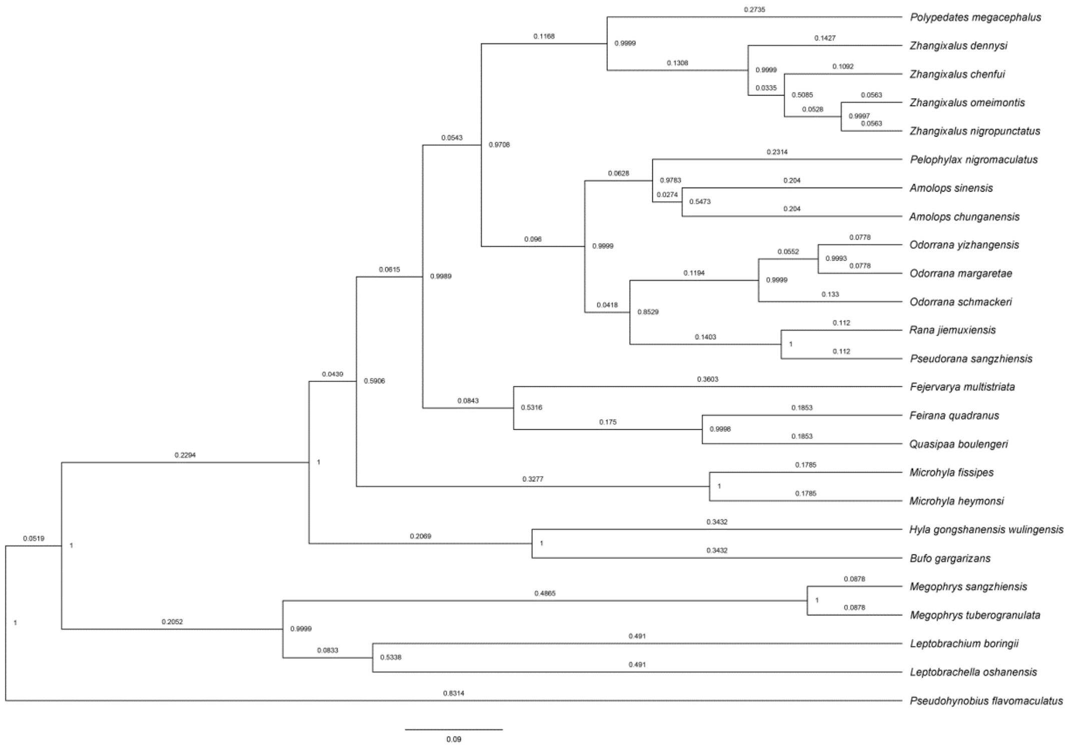

Figure S1. Phylogenetic tree of amphibian species in the study area. The values in the node indicated posterior probabilities.
